# Explainable AI to improve acceptance of convolutional neural networks for automatic classification of dopamine transporter SPECT in the diagnosis of clinically uncertain parkinsonian syndromes

**DOI:** 10.1007/s00259-021-05569-9

**Published:** 2021-10-15

**Authors:** Mahmood Nazari, Andreas Kluge, Ivayla Apostolova, Susanne Klutmann, Sharok Kimiaei, Michael Schroeder, Ralph Buchert

**Affiliations:** 1 Faculty of Computer Science and Center for Molecular and Cellular Bioengineering, Technical University Dresden, BiotechDresden, Germany; 2 ABX-CRO Advanced Pharmaceutical Services Forschungsgesellschaft M.B.H, 01307 Dresden, Germany; 3 Department of Diagnostic and Interventional Radiology and Nuclear Medicine, University Medical Center Hamburg-Eppendorf, Martinistr. 52, 20246 Hamburg, Germany; 4 Center for Molecular and Cellular Bioengineering, Technical University Dresden, Dresden, Germany

**Keywords:** Convolutional neural network, Explainable AI, Relevance propagation, Parkinson’s disease, Dopamine transporter, SPECT

## Abstract

**Purpose:**

Deep convolutional neural networks (CNN) provide high accuracy for automatic classification of dopamine transporter (DAT) SPECT images. However, CNN are inherently black-box in nature lacking any kind of explanation for their decisions. This limits their acceptance for clinical use. This study tested layer-wise relevance propagation (LRP) to explain CNN-based classification of DAT-SPECT in patients with clinically uncertain parkinsonian syndromes.

**Methods:**

The study retrospectively included 1296 clinical DAT-SPECT with visual binary interpretation as “normal” or “reduced” by two experienced readers as standard-of-truth. A custom-made CNN was trained with 1008 randomly selected DAT-SPECT. The remaining 288 DAT-SPECT were used to assess classification performance of the CNN and to test LRP for explanation of the CNN-based classification.

**Results:**

Overall accuracy, sensitivity, and specificity of the CNN were 95.8%, 92.8%, and 98.7%, respectively. LRP provided relevance maps that were easy to interpret in each individual DAT-SPECT. In particular, the putamen in the hemisphere most affected by nigrostriatal degeneration was the most relevant brain region for CNN-based classification in all reduced DAT-SPECT. Some misclassified DAT-SPECT showed an “inconsistent” relevance map more typical for the true class label.

**Conclusion:**

LRP is useful to provide explanation of CNN-based decisions in individual DAT-SPECT and, therefore, can be recommended to support CNN-based classification of DAT-SPECT in clinical routine. Total computation time of 3 s is compatible with busy clinical workflow. The utility of “inconsistent” relevance maps to identify misclassified cases requires further investigation.

**Supplementary Information:**

The online version contains supplementary material available at 10.1007/s00259-021-05569-9.

## Introduction

There is growing interest in the use of machine learning techniques for automatic classification of medical brain images to support the diagnosis of psychiatric and neurological diseases [1, 2]. Fully data-driven approaches based on deep convolutional neural networks (CNN) are particularly promising for this task [3]. CNN usually work end-to-end with no human knowledge built in, that is, without prior feature extraction (“image in, classification out”). The CNN itself learns the relevant features from a sufficiently large number of training cases with given standard-of-truth label (the clinical diagnosis after sufficiently long follow-up, for example). Deep CNN outperform conventional machine learning methods in many medical image classification tasks [4].

However, deep CNN are inherently black-box in nature so that improvement of classification accuracy by deep CNN comes at the price of reduced transparency. The multilayer nonlinear structure of CNN makes it difficult to identify the features automatically learned by the CNN during the training phase [5]. Furthermore, it is difficult to comprehend the basis of the CNN’s classification decision in new individual cases [5]. The lack of transparency is a major limitation of deep CNN, particularly in medical applications which require a human readable explanation of the automatic classification decision in each individual patient that allows the physician to verify that the classification decision made by the algorithm is plausible and coherent. The lack of transparency of deep CNN therefore limits their acceptance for widespread clinical use.

Recently developed techniques, called “explainable artificial intelligence,” aim at making CNN-based classification comprehensible for the user. Layer-wise relevance propagation (LRP) is an explainable AI technique that allows generation of an individual relevance map for each individual patient [6]. It relies on the application of deep Taylor decomposition and Kirchoff’s conservation law to the fully trained CNN for layer-wise backprojection of relevance starting from the most activated output neuron to the input layer [7]. The general concept of LRP is to build a local redistribution rule that is applied in a backward pass manner to each neuron. Different redistribution rules have been described for LRP [7, 8]. The individual relevance map generated by LRP is in the same space (with the same matrix) as the patient’s image used as input for the CNN. The voxel intensities in the relevance map indicate the relevance of the voxels for the CNN-based classification of this image [9]. In particular, the voxels in the input image that were most relevant for the CNN’s classification decision are identified by the highest intensity in the relevance map.

Here, we propose LRP with a specific combination of different redistribution rules in different parts of the CNN to explain CNN-based classification of single-photon emission computed tomography (SPECT) images of the dopamine transporter (DAT) availability in the brain of patients with a clinically uncertain parkinsonian syndrome.

## Materials and methods

### DAT-SPECT data

The PACS of the Department of Nuclear Medicine of the University Medical Center Hamburg Eppendorf was searched using the following inclusion criteria: (I1) DAT-SPECT had been performed to support the diagnosis of a clinically uncertain parkinsonian syndrome, (I2) DAT-SPECT had been performed with a double head SPECT system equipped with low-energy-high-resolution parallel-hole collimators according to standard procedure guidelines [10], and (I3) raw projection data were digitally available for consistent retrospective image reconstruction. No exclusion criteria were applied. This resulted in the inclusion of 1306 DAT-SPECT.

The projection data were reconstructed to tomographic SPECT images using filtered backprojection and a Shepp-Logan filter with cutoff 1.25 cycles/cm [11]. Neither attenuation correction nor scatter correction was applied [12]. Image reconstruction was performed using the “iradon” function of MATLAB (www.mathworks.com). All 1306 projection data were reconstructed fully automatically in a single batch using a custom MATLAB script in order to avoid errors by manual interaction.

Individual SPECT images were transformed (affine) into the anatomical space of the Montreal Neurological Institute (MNI) using the Statistical Parametric Mapping software package (version SPM12) [13] and a custom-made FP-CIT template. Voxel intensities were scaled to the 75^th^ percentile in a reference region comprising whole-brain except striata, thalamus, brain stem, and ventricles [14, 15].

The DAT-SPECT images were classified as “negative” (normal DAT-SPECT) or “positive” (reduced striatal tracer uptake typical for nigrostriatal degeneration in neurodegenerative parkinsonian syndromes) by two experienced readers based on visual inspection of a standardized display of the stereotactically normalized SPECT images [16]. Both readers had more than 10 years of experience in clinical reading of DAT-SPECT (200–400 cases per year). Each reader classified all images twice, blinded for all clinical information. Images with intra-reader discrepancy between the two reading sessions were assessed a third time by the same reader to obtain an intra-reader consensus. The resulting intra-reader consensus was in agreement between the two independent readers in 1275 of the 1306 cases (97.6%; Cohen’s kappa = 0.952 with standard error 0.008, *p* < 0.0005). The remaining 31 DAT-SPECT (2.4%), in which the intra-reader consensus differed between the two readers, were assessed in a common reading session of the two readers to obtain an inter-reader consensus. The latter was used as standard-of-truth in the further analyses. Ten of the 31 DAT-SPECT with discrepant intra-reader consensus showed an atypical striatal reduction pattern most likely caused by vascular/structural pathology and therefore were excluded (e.g., defect of FP-CIT uptake in the caudate nucleus with normal putaminal FP-CIT uptake, or complete lack of FP-CIT uptake in the whole striatum in one hemisphere with normal striatal FP-CIT uptake in the other hemisphere). The remaining 1296 DAT-SPECT were included in the study.

Visual inter-reader consensus read was “negative” in 676 (52.2%) of these DAT-SPECT; it was “positive” in the remaining 620 (47.8%) DAT-SPECT. This proportion of negative to positive cases (52.2 to 47.8%) is in line with the common recommendation to refer only patients with a clinically uncertain parkinsonian syndrome (CUPS) to DAT-SPECT [17], as “clinically uncertain” implies a pre-test probability of nigrostriatal degeneration of about 50%. The patient sample included in this study therefore can be considered representative of clinical routine according to common guidelines.

Clinical follow-up was not available in the vast majority of the included patients. From the subsample of patients in whom clinical follow-up was available, it might be assumed that amongst the patients with positive DAT-SPECT, about 90% had a disease from the spectrum of Lewy body diseases (Parkinson’s disease without and with cognitive impairment, dementia with Lewy bodies) whereas the remaining 10% suffered from an atypical neurodegenerative Parkinsonian syndrome including multiple system atrophy, progressive supranuclear palsy, and corticobasal degeneration [18]. The diagnoses of the patients with negative DAT-SPECT most likely included essential tremor, drug-induced parkinsonism, various types of dystonia, psychogenic parkinsonism, and various other diagnoses not associated with nigrostriatal degeneration [18].

### Image preprocessing for automatic classification

Specific FP-CIT binding to the DAT in the unilateral putamen in both hemispheres was characterized by the specific FP-CIT binding ratio estimated by hottest voxels analysis as described in the Supplementary Information (section “Conventional semi-quantitative analysis”). Stereotactically normalized DAT-SPECT images in which the putaminal specific binding ratio was lower in the right hemisphere were left–right mirrored at the midsagittal plane such that the putaminal specific binding ratio was lower in the left hemisphere in all cases. This was done in order to eliminate variability of no interest prior to automatic classification, since visual interpretation of the DAT-SPECT as standard-of-truth did not account for laterality (and was blinded for all clinical information, including laterality of motor symptoms). In the following, “ipsilateral” and “contralateral” (to the hemisphere with lower specific FP-CIT binding ratio in the putamen) are used instead of “left” and “right” hemispheres.

### Convolutional neural network

**Fig. 1:**
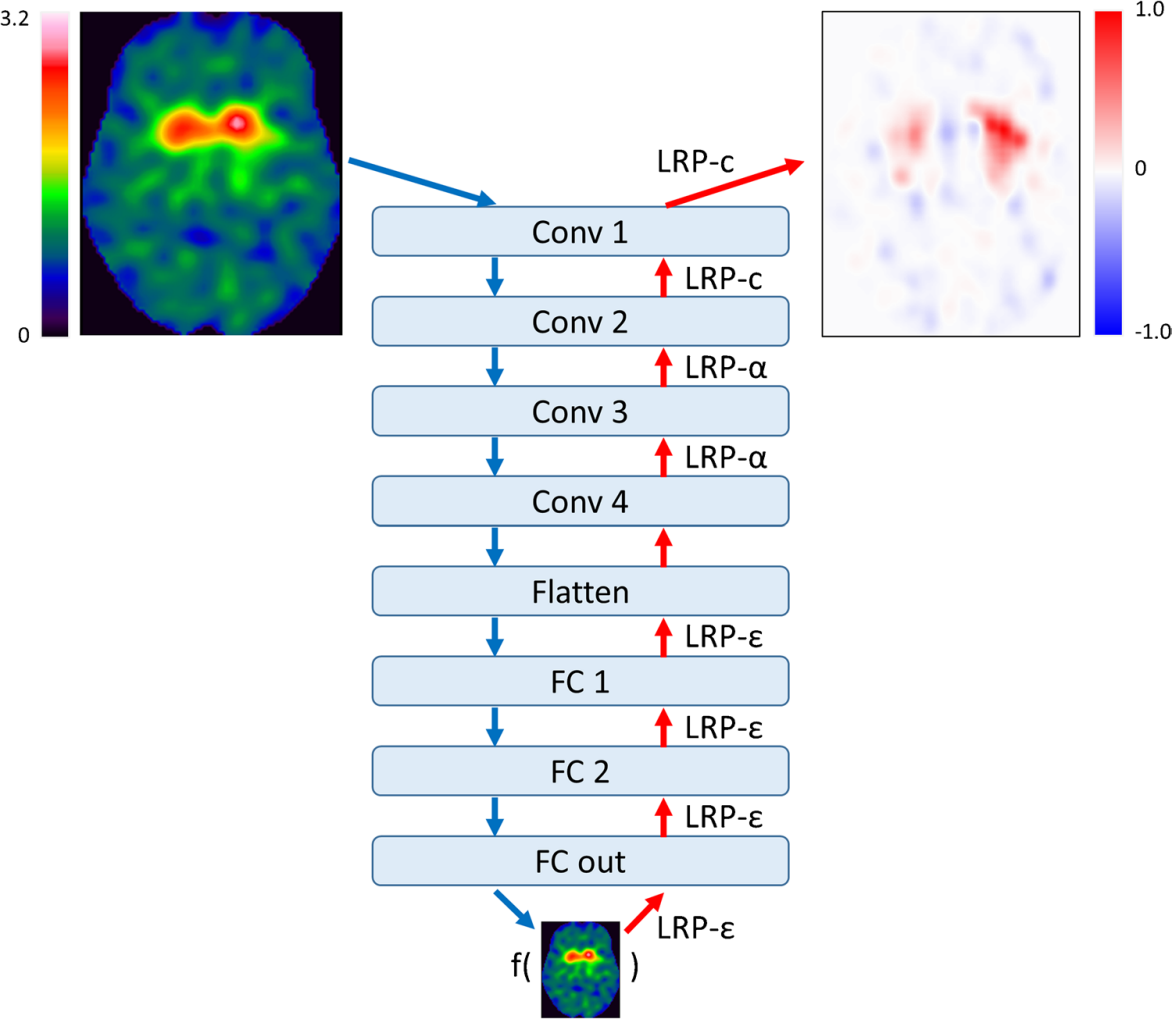
Structure of the custom CNN for binary classification of DAT-SPECT images. The LRP backprojection rule used at the different CNN layers to generate the relevance map (top right) corresponding to the CNN-based classification (bottom) of the DAT-SPECT (top left) is given at the red arrows. (Conv, convolutional layer; FC, fully connected layer)

The custom CNN trained for automatic classification of DAT-SPECT is shown in Fig. 1. It comprised four 3-dimensional convolutional layers with 16 filters, kernel size of 3 × 3 × 3. Stride and dilation were set to 1. The convolutional layers were followed by two fully connected neuron layers of 32 and 16 neurons, respectively, followed by a 2-way softmax output layer for binary classification. The rectified linear unit was used as activation function at all hidden layers. No pooling layers were used, mainly because all input images were in MNI space so that translation invariance was not required, but also to achieve a simple form of routing which routes all the features in the lower layer to the higher layer [19]. Drop out (0.2) was implemented in the first fully connected layer only. The total number of trainable CNN parameters was 236 million.

From the whole set of 1296 DAT-SPECT, two-thirds (*n* = 864) were randomized into the training set for the CNN. Allocating two-thirds of cases for training is recommended if the size of the whole dataset is reasonable (*n* ≥ 100) and if the expected accuracy of the classifier is good (≥ 85%) [20]. From the remaining one-third of the DAT-SPECT (*n* = 432), one-third (*n* = 144) was randomized into the validation set, two-thirds (*n* = 288) into the test set. The rationale for choosing the validation set smaller than the test set was that the validation set was only used to check for overfitting during the CNN training. The validation set was not used to compare different CNN designs, since only a single predefined CNN design was used in this study. A test set of size n allows estimation of the overall accuracy of the CNN for binary classification of DAT-SPECT with a maximum marginal error *d* at the 95% confidence level given by *d* = 1.96 * sqrt(acc*[1-acc])/sqrt(*n*), where acc is the expected accuracy [21]. Assuming acc = 0.9, the maximum marginal error of the overall accuracy of the CNN for binary classification of DAT-SPECT estimated from a test set of size *n* = 288 is 0.03. This appeared adequate for this study, because the primary aim was not to evaluate a specific CNN for automatic classification of DAT-SPECT but rather to evaluate LRP for the explanation of CNN-based classification of individual DAT-SPECT.

Randomization into training, validation, and test set was performed separately for females with negative DAT-SPECT (according to the inter-reader consensus), males with negative DAT-SPECT, females with positive DAT-SPECT, and males with positive DAT-SPECT, in order to achieve the same proportions of these four subgroups in training, validation, and test set. In order to achieve a similar age distribution in training, validation, and test set, separately for each of these four subgroups, a total of 100 random splits were generated, from which the random split with the minimum difference in mean age between training, validation, and test set over the four subgroups was selected for the analyses. Demographics in this random split are given in Table 1.

**Table 1:** Demographics in the whole sample of DAT-SPECT and in the random split for training, validation, and testing of the CNN. The age is given as mean value ± standard deviation in the subset

	Negative DAT-SPECT		Positive DAT-SPECT	
Age	Females	Males	Females	Males
Whole sample (*n* = 1296)	67.7 ± 11.3 (*n* = 296)	68.7 ± 11.6 (*n* = 380)	66.7 ± 11.0 (*n* = 246)	66.6 ± 11.0 (*n* = 374)
Training set (*n* = 864)	67.6 ± 11.4 (*n* = 197)	68.7 ± 11.9 (*n* = 254)	66.7 ± 11.2 (*n* = 164)	66.4 ± 10.8 (*n* = 249)
Validation set (*n* = 144)	68.2 ± 12.2 (*n* = 33)	68.3 ± 9.8 (*n* = 42)	66.8 ± 10.1 (*n* = 27)	66.8 ± 11.9 (*n* = 42)
Test set (*n* = 288)	67.6 ± 10.5 (*n* = 66)	68.8 ± 11.3 (*n* = 84)	66.7 ± 11.1 (*n* = 55)	66.9 ± 11.0 (*n* = 83)

The CNN was trained with a batch size of 8 against the categorical cross-entropy loss using the Adam optimizer with 10^−4^ learning rate. Loss weighting for different classes was not used, because the data were balanced with respect to the class to good approximation.

Using an Nvidia Titan XP graphic card with 12 GB memory, the training of the CNN took approximately 64 s per epoch. The CNN could be trained without noticeable overfitting. The total training time until convergence was approximately 1.5 h.

### Layer-wise relevance propagation

**Fig. 2:**
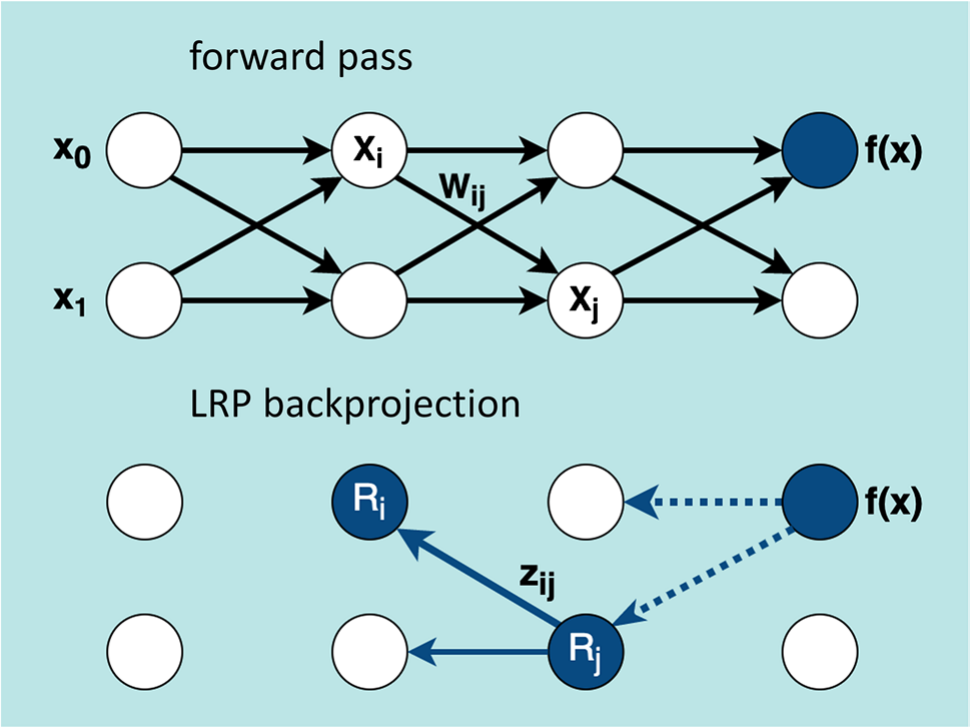
LRP relevance backprojection. The neural network (top) with the trained weights *w*_*ij*_ is used in forward pass to calculate the output score *f*(*x*) for the given input *x* = *(x*_*0*_*, x*_*1*_*)*. In LRP (bottom), the neuron *R*_*i*_ receives the relevance *z*_*ij*_ from the higher-level layer neuron *R*_*j*_ (solid arrow). The dotted arrows indicate the relevance flow into the layer containing the neuron *R*_*j*_ calculated previously. The flow starts from the most activated output neuron

In order to estimate the relevance of each single voxel of the subject’s image for the classification of the whole image by the CNN, LRP takes advantage of the CNN graph structure for layer-wise backprojection of relevance from the most activated output neuron up to the input layer (Fig. 1) [6, 22]. More precisely, LRP is based on a local backprojection rule to redistribute relevance from neurons in a given layer to the neurons in the preceding layer as illustrated in Fig. 2. If *z*_*ij*_ denotes the fraction of the relevance ${R}_{j}^{\left[k\right]}$ at neuron *j* in the CNN layer *k* that is backprojected to neuron *i* in the preceding layer *k* − 1, then the total relevance ${R}_{i}^{\left[k-1\right]}$ at neuron *i* is given by

1 $${R}_{i}^{[k-1]}=\sum_{j\in [k]}\frac{{z}_{ij}}{{\sum }_{i\in [k-1]}{z}_{ij}}{R}_{j}^{[k]}$$

The scaling factors $\sum_{i\in [k-1]}{z}_{ij}$ in the denominator on the right-hand side guarantee that the relevance is preserved during backprojection at each neuron. When the rectified linear unit is used as activation function, first-order Taylor expansion at the prediction point suggests the following standard choice for the backprojection coefficients [7]

2 $${z}_{ij}={a}_{i} {w}_{ij}$$

where ${a}_{i}$ is the activation of neuron *i* for the considered image in the prediction phase (forward pass) and ${w}_{ij}$ is the weight factor for the input to neuron *j* from neuron *i* fixed during the training phase (Fig. 2).

Several variations of the LRP rule according to Eqs. 1 and 2 have been proposed [7, 8]. In the present study, three of these variations were combined for (i) improved robustness of LRP by avoiding noise amplification due to the gradient shattering effect [23, 24], (ii) reduced spill-out of relevance, and (iii) discrimination between features that support the prediction and features that oppose it.

The propagation rule

3 $$\mathrm{LRP}-\upvarepsilon : {R}_{i}^{[k-1]}=\sum_{j\in [k]}\frac{{z}_{ij}}{{\sum }_{i\in [k-1]}\left\{{z}_{ij}+ \epsilon sign\left({z}_{ij}\right)\right\}}{R}_{j}^{[k]}$$

with *z*_*ij*_ according to Eq. 2 was used for relevance backprojection at the fully connected layers close to the output of the CNN (Fig. 1). Here, *sign(x)* denotes the sign of *x*, that is, *sign(x)* = 1 for *x* ≥ 0 and *sign(x)* =  − 1 for *x* < 0. The ε-term is introduced to limit noise amplification. ε = 0.0001 was used.

The propagation rule

4 $${\mathrm{LRP}-{\alpha }: R}_{i}^{[k-1]}=\sum_{j\in [k]}(\alpha \frac{{z}_{ij}^{+}}{{\sum }_{i\in [k-1]}{\mathrm{z}}_{ij}^{+}}+\left(\alpha -1\right)\frac{{z}_{ij}^{-}}{{\sum }_{i\in [k-1]}{\mathrm{z}}_{ij}^{-}})$$

with *z*_*ij*_ according to Eq. 2 was used for relevance backprojection at the fourth and the third convolutional layers (Fig. 1). Here, “ + ” and “ − ” indicate the positive and the negative parts, respectively, that is

5a $${z}_{ij}^{+}=\mathrm{max}(0,{z}_{ij})$$

5b $${z}_{ij}^{-}=\mathrm{min}(0,{z}_{ij})$$

The parameter *α* was chosen as *α* = 2 in order to allow for both positive and negative relevance. Positive relevance indicates that the feature supports the classification decision whereas negative relevance indicates that the feature provides evidence against it.

Finally, uniform backprojection (LRP-c) defined by Eq. 1 with *z*_*ij*_ = 1 was used at the first two layers close to the input of the CNN for improved control of resolution and semantics in the relevance maps [25] (Fig. 1).

### Statistical analysis

The classification performance of the CNN was estimated in the test set (independent of the training set) in order to avoid overly optimistic performance estimates due to overfitting. Overall accuracy, sensitivity specificity, and predictive values were used to characterize classification performance.

The relevance maps generated by LRP were assessed visually for each DAT-SPECT in the test set in order to evaluate their interpretability.

## Results

CNN-based classification in the test set resulted in 148 true negative cases, 128 true positive cases, ten false negative cases, and two false positive cases. Thus, overall accuracy, sensitivity, specificity, positive, and negative predictive values of the CNN for classification of the DAT-SPECT in the test set were 95.8%, 92.8%, 98.7%, 98.5%, and 93.7%, respectively. The CNN performance was similar to the performance of conventional semi-quantitative analysis and of classification and regression tree analysis (Supplementary Information).

**Fig. 3:**
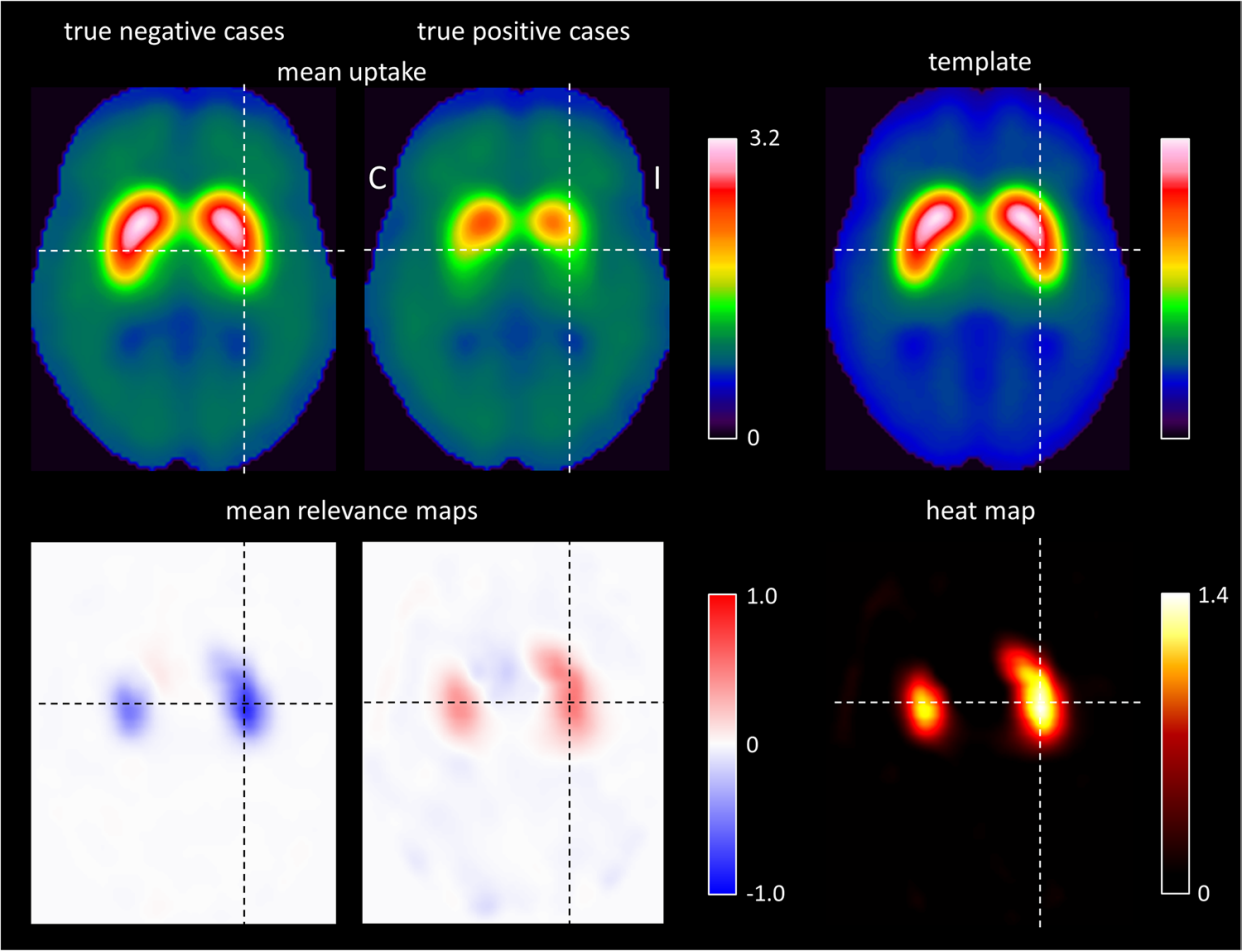
Representative transaxial slice through the striatum of the mean DAT-SPECT image (top row) and of the mean relevance map (bottom row) in negative (left column) and positive (middle column) cases correctly classified by the CNN. All slices of the mean relevance maps are shown in supplementary Fig. 1. The right column shows the custom-made DAT-SPECT template used for stereotactical normalization (top) and the heat map defined as the difference of the mean relevance map in true positive cases minus the mean relevance map in true negative cases (bottom). (I / C, Ipsilateral / Contralateral to the hemisphere with lower specific FP-CIT binding ratio in the putamen)

A representative transaxial slice of the mean relevance map is shown in Fig. 3, separately for the true negative and the true positive DAT-SPECT (all transaxial slices of the mean relevance maps are given in supplementary Fig. 1). The mean relevance map of the true negative cases was the inverse (sign flip) of the mean relevance map of the true positive cases to good approximation. This suggested the computation of a “heat map” by computation of the voxel-based difference of the mean relevance map of the true negative cases minus the mean relevance map of true positive cases in order to simplify identification of the brain regions with the highest relevance for the CNN-based classification (Fig. 3). The ipsilateral putamen (with the strongest reduction of FP-CIT uptake in the positive cases) showed the highest relevance (heat) followed by the contralateral putamen and the ipsilateral caudate nucleus (Fig. 3). The most relevant single voxel was located in the striatum (or very close) in all cases.

**Fig. 4:**
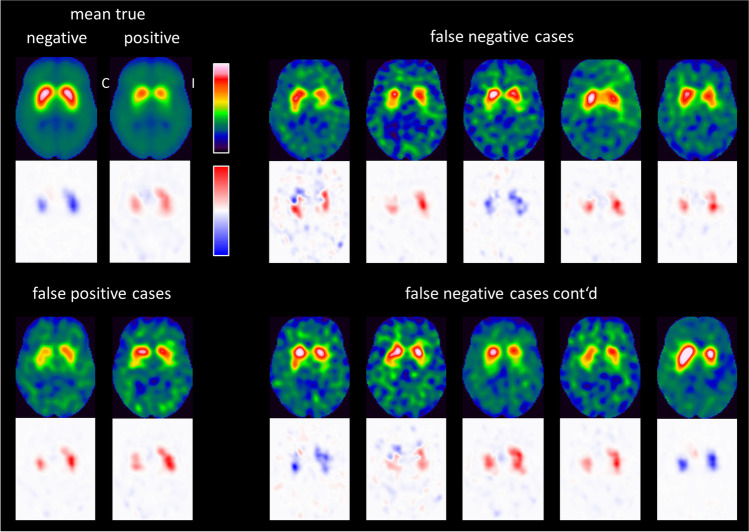
Individual relevance maps of the 12 amongst the 288 test cases that were misclassified by the CNN. The mean DAT-SPECT and the mean relevance map in true negative and true positive cases (from Fig. 3) are shown for comparison. (I / C, Ipsilateral / Contralateral to the hemisphere with lower specific FP-CIT binding ratio in the putamen)

**Fig. 5:**
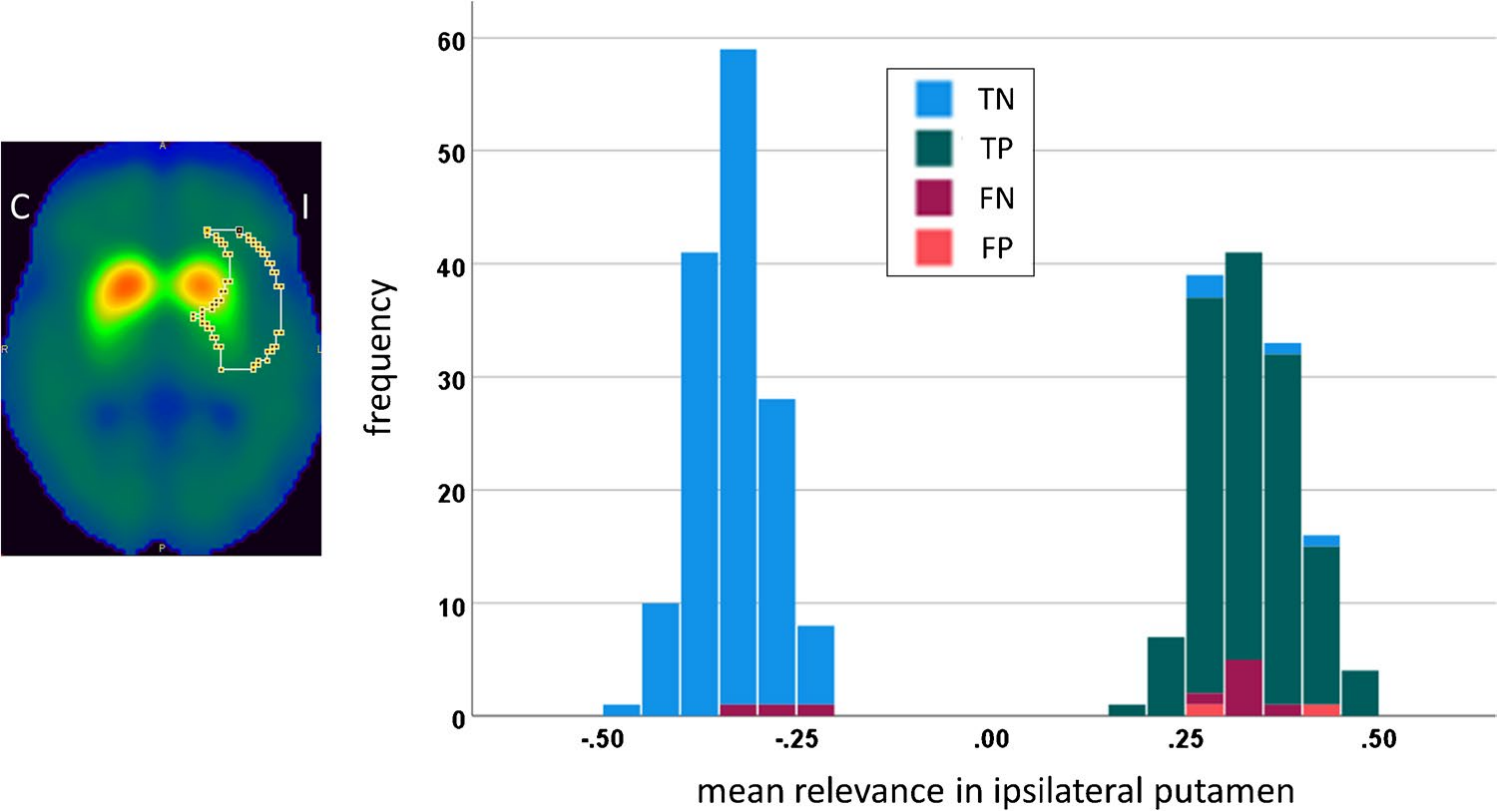
Outer contour of the large putamen ROI used to compute the mean relevance in the ipsilateral putamen by hottest voxels analysis (left). The ROI is overlaid to the mean DAT-SPECT of the true negative cases. The right part shows the histogram of the mean relevance of the ipsilateral putamen in the test set. The color indicates the CNN-based classification (TN, true negative; TP, true positive; FN, false negative; FP, false positive; I / C, Ipsilateral / Contralateral to the hemisphere with lower specific FP-CIT binding ratio in the putamen)

Figure 4 shows the individual relevance maps of the DAT-SPECT misclassified by the CNN. The two false positive DAT-SPECT showed borderline FP-CIT uptake in the striatum so that the standard-of-truth label might be questioned and the CNN-based classification might actually be correct in these cases. The ten false negative DAT-SPECT all presented clear reduction of the FP-CIT uptake in the ipsilateral putamen (in line with the standard-of-truth) indicating that they were actually misclassified by the CNN. It is striking that seven of the ten false negative cases showed an “inconsistent” relevance map with positive relevance in the striatal region, most pronounced in the ipsilateral putamen, which is typical for true positive cases. This suggests that the striatal signal in the relevance maps might be implemented to improve the classification accuracy. In order to test this, the mean relevance in the ipsilateral putamen was determined for all DAT-SPECT in the test set. The same hottest voxels analysis was used for this purpose as for the estimation of the putaminal specific FP-CIT binding ratio (Supplementary Information). The distribution of the mean relevance in the ipsilateral putamen in the test set is shown in Fig. 5. When the mean relevance in the ipsilateral putamen was dichotomized with cutoff zero and then used for classification of the DAT-SPECT (negative and positive mean relevance in the ipsilateral putamen indicating negative and positive DAT-SPECT, respectively), it provided very similar performance as the CNN-based classification (overall accuracy, sensitivity, specificity, positive, and negative predictive values of 96.9%, 97.8%, 96.0%, 95.7%, and 98.0%, respectively).

## Discussion

Deep CNN are increasingly used for automated classification of medical images to assist the physician in their interpretation [4]. They are however black-box in nature, that is, they do not provide any kind of explanation for their decisions, in contrast to many conventional classification methods, e.g., decision trees. This makes it difficult to identify their mechanism of making decisions and to comprehend their decision in individual cases. This limits the acceptance of deep CNN for widespread clinical use. Recent efforts to address this limitation, combined under the umbrella term “explainable AI”, resulted in the development of several methods to provide transparency of black-box models [26–29]. LRP is one of these new methods [6]. It allows tracking back the classification result from the output layer of the deep CNN to its input layer in order to generate an individual relevance map. The voxels with the highest relevance (highest absolute value) had the strongest impact on the CNN’s decision in this case. Thus, individual relevance maps allow the user to understand and check the CNN-based classification in individual patients. This is expected to improve acceptance of CNN-based classification for clinical use, provided that LRP works reliably in images from clinical routine. The present study tested this for DAT-SPECT to detect or exclude nigrostriatal degeneration in patients with clinically uncertain parkinsonian syndromes [17]. Previous LRP applications in medical brain imaging include MRI-based diagnosis of Alzheimer’s disease [9] and multiple sclerosis [30].

In DAT-SPECT, visual interpretation of the images by a trained physician is sufficient for clinical reporting in the majority of cases [31]. However, quantitative analysis and/or automatic classification is a useful adjunct when used as an objective second reader, particularly in borderline cases and for less experienced readers [32]. Conventional machine learning methods using support vector machines [33–43], decision trees [44, 45], or cluster analyses [46] based on a (small) set of predefined image-derived features have been proposed for this purpose. However, recent work suggests that artificial neural networks, particularly deep CNN, outperform conventional approaches for the automatic classification of DAT-SPECT [18, 47–58], partly because artificial neural networks can be less sensitive to camera- and site-specific variability of image quality (e.g., with respect to spatial resolution) [18]. Thus, deep CNN are very promising to support interpretation of DAT-SPECT in clinical routine so that there is a high clinical need for methods to explain CNN-based classification in individual patients.

The custom CNN used in the present study achieved high overall accuracy of 95.8%, in line with previous studies demonstrating excellent performance of artificial networks for automatic classification of DAT-SPECT [18, 47–58]. Specificity was somewhat higher than sensitivity. In order to test whether this is a characteristic of the custom CNN design and/or the patient sample used in this study, CNN training and testing was repeated several times (using the same random split for training, validation, and testing, but with different initialization of the CNN weights prior to the training). The overall accuracy was very similar in all repeats, but the ordering of sensitivity and specificity (“sensitivity > specificity” or “specificity > sensitivity”) varied between repeats (results not shown). This suggests that there was no bias in favor of sensitivity or specificity in this study.

LRP provided relevance maps that were easy to interpret in each individual patient, although the study did not impose specific eligibility criteria on the DAT-SPECT images. In particular, there were no requirements with respect to the total number of counts in order to restrict the analyses to images with high statistical image quality. This demonstrates that CNN-based classification and LRP are stable with respect to variability of the statistical quality of DAT-SPECT images encountered in clinical routine. This is an important requirement for widespread clinical use.

The putamen in the hemisphere most affected by nigrostriatal degeneration was identified as the most relevant brain region for CNN-based classification in each individual patient. Much less relevance was attributed to extrastriatal brain regions by LRP, in line with the fact that extrastriatal signal in DAT-SPECT most likely represents tracer binding to serotonin transporters (not dopamine transporters), which are relatively preserved in Parkinson’s disease [59].

The mean relevance map of true negative cases was very similar to the mean relevance map of the true positive cases except for a sign flip (Fig. 3). That the same image voxels are the most relevant independent of the class (negative or positive), is a specific characteristic of binary image classification tasks. In the present case, FP-CIT uptake in the ipsilateral putamen was the most prominent difference between negative and positive DAT-SPECT. Thus, it was to be expected that the CNN attributed the highest relevance to the ipsilateral putamen independent of the class: normal FP-CIT uptake in the ipsilateral putamen was the strongest indicator of a negative DAT-SPECT; reduced FP-CIT uptake in the ipsilateral putamen was the strongest indicator of a positive DAT-SPECT.

A few of the cases misclassified by the CNN showed an “inconsistent” relevance map (peak relevance values in the ipsilateral putamen with the “wrong” sign) more typical for the true classification, suggesting that individual relevance maps might be useful to identify misclassified cases. This requires further investigation, although re-classification of DAT-SPECT based on the ipsilateral putaminal signal in the individual relevance maps in this study provided some evidence for it.

The relevance map of an individual DAT-SPECT image is not intended to provide new insights into the pathophysiology of clinically uncertain parkinsonian syndromes but rather to explain the classification of the CNN for this DAT-SPECT image. However, on the group level, LRP might be useful to extract information from a trained CNN about extrastriatal signal in DAT-SPECT that might contribute to the differentiation between neurodegenerative and non-neurodegenerative etiologies. This might contribute to a better understanding of clinically uncertain parkinsonian syndromes.

Magesh and coworkers recently suggested the Local Interpretable Model-Agnostic Explainer (LIME) method to explain automatic classification of DAT-SPECT with the VGG16 network [60] adapted for this task by transfer learning [48]. The LIME method identifies “supervoxels” in the SPECT images for visual control. The authors concluded that the VGG16 network combined with LIME-based explanation is useful to support interpretation of DAT-SPECT [48].

The following limitation of this study should be noted. The CNN was trained to reproduce the visual interpretation of DAT-SPECT by experienced readers and, therefore, might not provide the correct etiological/biological diagnosis in all cases. We also do not claim that the specific CNN used in this study is superior to other CNN for the classification of DAT-SPECT described previously. However, the primary aim of this study was not to propose a specific CNN for automatic classification of DAT-SPECT but rather to evaluate layer-wise relevance propagation to explain CNN-based classification of DAT-SPECT in individual cases. LRP is a novel explainable AI technique. It is not restricted to the specific CNN used in the present study but it is easily implemented for other CNN with different structure (e.g., different number of layers).

In conclusion, layer-wise relevance propagation is useful to provide explanation of CNN-based decisions in individual DAT-SPECT and, therefore, can be recommended to support CNN-based classification of DAT-SPECT in clinical routine. Total computation time of 3 s is compatible with busy clinical workflow. The use of relevance maps to improve the classification by identifying misclassified cases requires further investigation.

## Supplementary Information

Below is the link to the electronic supplementary material.

Supplementary file1 (PDF 743 KB)

## Data Availability

The relevance maps generated during this study can be made available on request.

## Abbreviations

AI: Artificial intelligence
CNN: Convolutional neural network
DAT: Dopamine transporter
FP-CIT: N-ω-fluoropropyl-2β-carbomethoxy-3β-(4-^123^I-iodophenyl)nortropane
LIME: Local Interpretable Model-Agnostic Explainer
LRP: Layer-wise relevance propagation
MNI: Montreal Neurological Institute
SPECT: Single-photon emission computed tomography
SPM: Statistical parametric mapping
