# Supplementary material for: Explainable AI to improve acceptance of convolutional neural networks for automatic classification of dopamine transporter SPECT in the diagnosis of clinically uncertain parkinsonian syndromes
Source: Eur J Nucl Med Mol Imaging. 2021 Oct 15;49(4):1176–86. doi: 10.1007/s00259-021-05569-9 (PMC8921148; doi:10.1007/s00259-021-05569-9)
Supplement: Supplementary file 1 — Supplementary file1 (PDF 743 KB) [file 259_2021_5569_MOESM1_ESM.pdf]

Supplementary Information

Explainable AI to improve acceptance of convolutional neural networks for automatic classification of dopamine transporter SPECT in the diagnosis of clinically uncertain parkinsonian syndromes

Mahmood Nazari[1,2], Andreas Kluge[2], Ivayla Apostolova[3], Susanne Klutmann[3], Sharok Kimiaei[2], Michael Schroeder[1], Ralph Buchert[3]

[1]Department of Computer Science, Biotech, Technical University Dresden, Germany

[2]ABX - CRO advanced pharmaceutical services Forschungsgesellschaft m.b.H., D-01307 Dresden, Germany

[3]Department of Diagnostic and Interventional Radiology and Nuclear Medicine, University Medical Center Hamburg-Eppendorf, Hamburg, Germany

Conventional semi-quantitative analysis

Conventional region-of-interest (ROI)-based semi-quantitative analysis was performed using hottest voxels analysis as described previously [1-3]. In brief, large ROIs predefined in the anatomical standard space of the Montreal Neurological Institute (MNI) were applied to the scaled DAT-SPECT images after stereotactical normalization to MNI space to compute the specific FP-CIT binding ratio (SBR) in the putamen and in the caudate nucleus, separately in both hemispheres. The predefined ROIs were much bigger than the anatomical putamen and caudate in order to guarantee that these structures were completely included in the standard ROIs in each individual patient, independent of some residual anatomical inter-subject variability after stereotactical normalization. The number of hottest voxels to be averaged was fixed to a total volume of 10 ml for the unilateral putamen and 5 ml for the unilateral caudate. The total volume of 15 ml for caudate and putamen is compatible with the normal range of the striatum volume in healthy subjects [4]. The unilateral SBR in putamen or caudate was calculated as mean scaled (to the reference region) voxel intensity in the corresponding hottest ROI voxels – 1. From these, the following additional semi-quantitative parameters were derived: putamen-to-caudate SBR ratio in the left and in the right hemisphere, left-right asymmetry of putamen SBR and left-right asymmetry of caudate SBR (left-right asymmetry = 200*abs(left-right)/(left+right)). In order to eliminate variability of no interest associated with mainly left-sided versus mainly right-sided nigrostriatal degeneration (that might affect the performance of the unilateral semi-quantitative parameters), minimum and maximum of putamen and caudate SBR and of the putamen-to-caudate SBR ratio of both hemispheres were considered rather than left and right values. Thus, the following eight semi-quantitative parameters were tested for differentiation between positive and negative DAT-SPECT: minimum of left and right putamen SBR, maximum of left and right putamen SBR, minimum of left and right caudate SBR, maximum of left and right caudate SBR, minimum of left and right putamen-to-caudate SBR ratio, maximum of left and right putamen-to-caudate SBR ratio, left-right asymmetry of putamen SBR, and left-right asymmetry of caudate SBR.

The cutoff for each of these parameters was determined in the DAT-SPECT training set by the Youden criterion [5] applied to their receiver operating characteristic curve for identification of positive cases (supplementary Fig. 2). The cutoffs determined in the training set were then applied for classification of the DAT-SPECT images in the test set. Overall accuracy, sensitivity, specificity, positive predictive value and negative predictive value were determined as performance measures. Training set and test set were the same as for training and testing of the CNN.

The best overall accuracy by a single semi-quantitative parameter was achieved by the minimum putamen SBR (96.9%, supplementary Tab. 1), followed by the maximum putamen SBR (93.1%) and the minimum putamen-to-caudate SBR ratio (91.3%). Left-right asymmetry of putamen and caudate SBR achieved the lowest overall accuracy (80.2 and 81.6%) but still considerable above chance level (50%).

Classification and regression tree analysis

Classification and regression tree (CRT) analysis was tested for automatic classification of the DAT-SPECT included in this study. CRT analysis was selected as multivariable machine learning method, since not only the mechanism of the CRT learned during the training is particularly easy to understand for users but also its decision in individual cases. CRT analysis for the identification of positive DAT-SPECT included the eight semi-quantitative SBR parameters described in the supplementary section "Conventional semi-quantitative analysis" as continuous variables. The CRT was trained in the training set (same as for CNN training) using the chi-square automatic interaction detection technique. The depth of the tree was fixed to two levels, the minimum number of cases for parent/child nodes was set to 100/50. IBM SPSS Statistics version 27 was used for the CRT analysis.

The CRT selected the minimum putamen SBR for branching at the root level (supplementary Fig. 3). In the test set (same as for CNN testing), the proportion of positive DAT-SPECT increased from 47.9% in the whole test set to 92.5% in DAT-SPECT with reduced minimum putamen SBR, and it decreased to 1.4% in DAT-SPECT with relatively normal minimum putamen SBR. The maximum putamen-to-caudate SBR ratio was selected for second level branching of DAT-SPECT with reduced minimum putamen SBR. The proportion of positive DAT-SPECT further increased to 98.3% in the DAT-SPECT with reduced maximum putamen-to-caudate SBR ratio. In contrast, left-right asymmetry of the putamen SBR was selected for second level branching of DAT-SPECT with relatively normal minimum putamen SBR. The proportion of patients with positive DAT-SPET further decreased from 1.4% to 0% in the DAT-SPECT with more left-right symmetric putamen SBR. Overall classification accuracy in the test set was 95.5%. Thus, the CRT achieved about the same overall accuracy as the CNN (95.5% versus 95.8%). Further performance measures of the CRT are given in supplementary Tab. 1.

References to the supplementary material

1. Schmitz-Steinkruger H, Lange C, Apostolova I, Amthauer H, Lehnert W, Klutmann S, et al. Impact of the size of the normal database on the performance of the specific binding ratio in dopamine transporter SPECT. Ejnmmi Phys. 2020;7:34. doi:10.1186/s40658-020-00304-z.

2. Schmitz-Steinkruger H, Lange C, Apostolova I, Mathies FL, Frings L, Klutmann S, et al. Impact of age and sex correction on the diagnostic performance of dopamine transporter SPECT. Eur J Nucl Med Mol Imaging. 2021;48:1445-59. doi:10.1007/s00259-020-05085-2.

3. Wenzel M, Milletari F, Kruger J, Lange C, Schenk M, Apostolova I, et al. Automatic classification of dopamine transporter SPECT: deep convolutional neural networks can be trained to be robust with respect to variable image characteristics. Eur J Nucl Med Mol Imaging. 2019;46:2800-11. doi:10.1007/s00259-019-04502-5.

4. Aylward EH, Li Q, Habbak QR, Warren A, Pulsifer MB, Barta PE, et al. Basal ganglia volume in adults with Down syndrome. Psychiatry Res. 1997;74:73-82.

5. Youden WJ. Index for rating diagnostic tests. Cancer. 1950;3:32-5.

Supplementary Figures

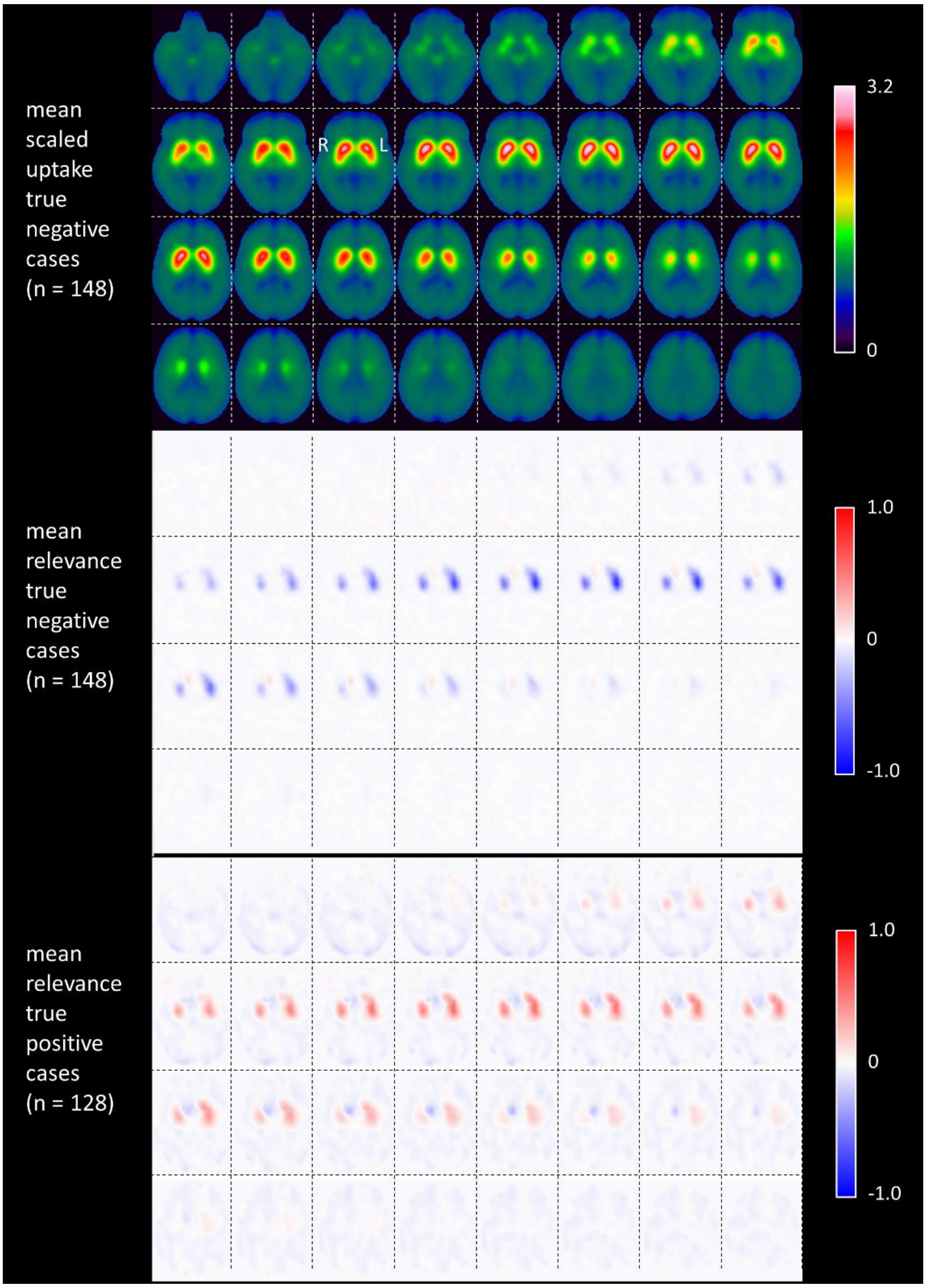

Supplementary Fig. 1 Transaxial slices of the mean DAT SPECT image in the negative cases (top) and of the mean relevance map in true negative cases (middle) and in true positive cases (bottom)

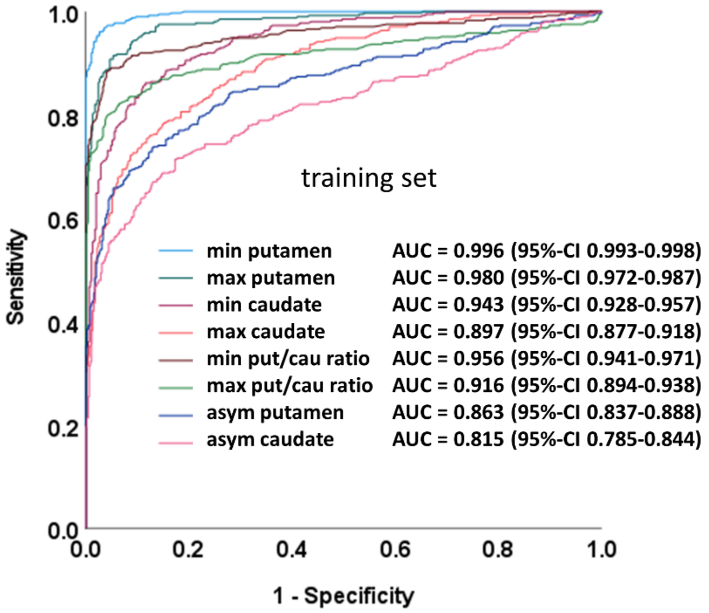

Supplementary Fig. 2 Receiver operating characteristic curves for identification of positive DAT-SPECT by conventional semi-quantitative SBR parameters in the training set.

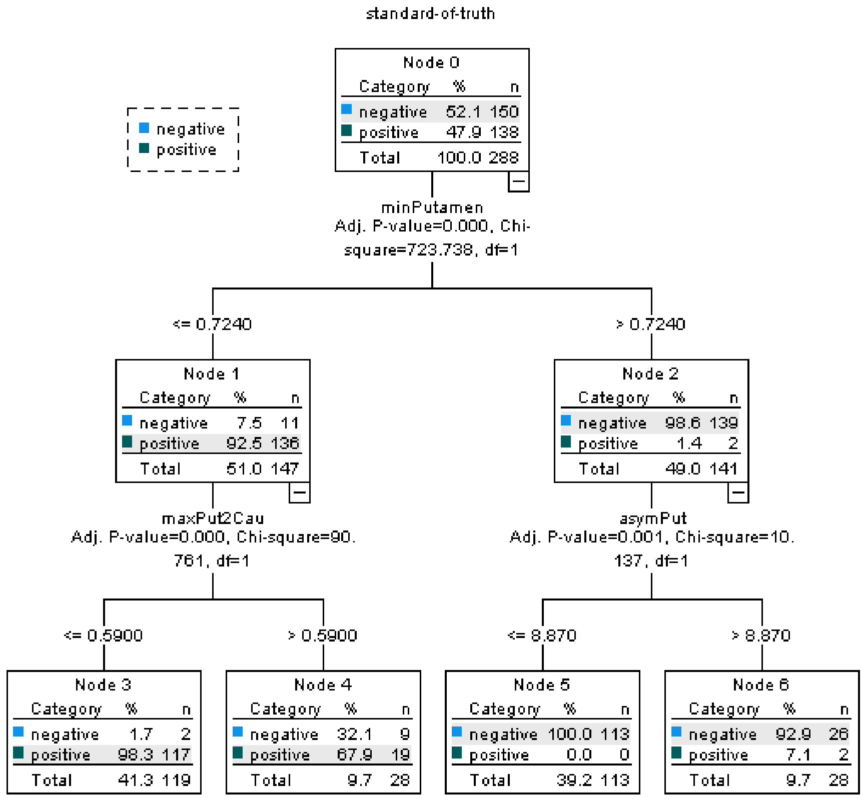

Supplementary Fig. 3 Classification and regression tree performance in the test set. The tree was trained in the training set. Training and test set were the same as in the CNN analyses.

Supplementary Tables

Supplementarty Tab. 1 Performance of the semi-quantitative SBR parameters in the test set in the univariate analyses. The cutoffs were determined in the training set. Performance of the multivariate machine learning methods in the test set is given for comparison. (acc = overall accuracy, sens = sensitivity, spec = specificity, PPV = positive predictive value, NPV = negative predictive value, SBR = specific FP-CIT binding ratio, CRT = classification and regression tree, CNN = convolutional neural network)

univariate models	acc [%]	sens [%]	spec [%]	PPV [%]	NPV [%]
minimum putamen SBR	96.9	97.1	96.7	96.4	97.3
maximum putamen SBR	93.1	90.6	95.3	94.7	91.7
minimum caudate SBR	86.5	84.1	88.7	87.2	85.8
maximum caudate SBR	82.3	75.4	88.7	86.0	79.6
minimum putamen-to-caudate SBR ratio	91.3	85.5	96.7	95.9	87.9
maximum putamen-to-caudate SBR ratio	85.8	77.5	93.3	91.5	81.9
left-right asymmetry putamen SBR	80.2	68.1	91.3	87.9	75.7
left-right asymmetry caudate SBR	81.6	81.2	82.0	80.6	82.6
**multivariable models**	**acc [%]**	**sens [%]**	**spec [%]**	**PPV [%]**	**NPV [%]**
CRT	95.5	98.6	92.7	92.5	98.6
CNN	95.8	92.8	98.7	98.5	93.7
relevance in ipsilateral putamen	96.9	97.8	96.0	95.7	98.0
